# Supplementary material for: Identification and functional analysis of non-coding regulatory small RNA FenSr3 in Bacillus amyloliquefaciens LPB-18
Source: PeerJ. 2023 May 15;11:e15236. doi: 10.7717/peerj.15236 (PMC10194069; doi:10.7717/peerj.15236)
Supplement: Supplemental Information 4 [file peerj-11-15236-s004.zip › KO/CK-vs-T1_map/map04722.html]

KEGG PATHWAY: Neurotrophin signaling pathway - Reference pathway


|  |  |
| --- | --- |
| **Neurotrophin signaling pathway - Reference pathway** |  |

[
Pathway menu
| Organism menu
| Pathway entry
| Show description
| User data mapping
]

Neurotrophins are a family of trophic factors involved in differentiation and survival of neural cells. The neurotrophin family consists of nerve growth factor (NGF), brain derived neurotrophic factor (BDNF), neurotrophin 3 (NT-3), and neurotrophin 4 (NT-4). Neurotrophins exert their functions through engagement of Trk tyrosine kinase receptors or p75 neurotrophin receptor (p75NTR). Neurotrophin/Trk signaling is regulated by connecting a variety of intracellular signaling cascades, which include MAPK pathway, PI-3 kinase pathway, and PLC pathway, transmitting positive signals like enhanced survival and growth. On the other hand, p75NTR transmits both positive and nagative signals. These signals play an important role for neural development and additional higher-order activities such as learning and memory.

|  |  |  |
| --- | --- | --- |
| Reference pathway | 184% 150% 122% 100% 82% 67% 55% | 图片下载 |
